# Accuracy of Stool Antigen Test in the Diagnosis of Helicobacter pylori Infection in the Dominican Republic

**DOI:** 10.7759/cureus.44290

**Published:** 2023-08-28

**Authors:** Miguel Alfau, Annerys Delgado, Cinthia Reyes, Diana Durán, Diego Arbaje, Annette García

**Affiliations:** 1 Gastroenterology and Hepatology, Instituto Materno Infantil y Especialidades, Santiago, DOM; 2 Pathology, Instituto Materno Infantil y Especialidades, Santiago, DOM; 3 School of Medicine, Pontificia Universidad Católica Madre y Maestra, Santiago, DOM

**Keywords:** specificity, sensitivity, gastric biopsy, stool antigen test, helicobacter pylori

## Abstract

Introduction*: Helicobacter pylori* is a well-studied infectious agent due to its pathogenic potential for peptic ulcers and gastric cancer. It has a high prevalence worldwide and has several diagnostic methods, both invasive and non-invasive. It is important to address the diagnostic efficacy of these tests, as the data vary by location and the specific population in which they are used. Therefore, an effective testing method should be obtained, evaluating the possibility of substantially reducing invasive procedures and, therefore, associated costs.

Objective: This study proposes to define the diagnostic accuracy of the stool antigen test for *H. pylori* infection in the Dominican Republic.

Methods: An observational, retrospective, and cross-sectional study was conducted. The results of the stool antigen test for* H. pylori *infection were compared with the results of the gastric biopsy, as a gold standard test. Patients over 18 years of age with an indication for endoscopy due to suspicion of *H. pylori* infection, who attended the gastroenterology clinic in 2021, were included in the study.

Results: It was shown that the stool antigen test for *H. pylori* infection has a 61.54% sensitivity and 59.65% specificity. According to the study population, the positive predictive value (PPV) was 67.60% and the negative predictive value (NPV) was 53.13%.

Conclusion: Low numbers of both sensitivity and specificity were determined, which is why it is pertinent to study alternative non-invasive methods. However, it is important to assess the antibiotic exposure of the study population, since the diagnostic accuracy of the stool test can be influenced by this factor.

## Introduction

*Helicobacter pylori* is a well-studied cause of gastritis worldwide. This pathogen is present in 50% of the population [1]. There is a well-established socioeconomic relationship with this microorganism, which is much more common in developing countries than in more developed countries [1]. In this sense, the presence of *H. pylori* in adults from Latin American countries varies, with rates of 65% in Guatemala, 75-90% in Mexico and Chile, and 82% in Brazil [1]. In the Dominican Republic, the estimated prevalence was 84% in 2006 [2]. In the United States, a prevalence of 56.4% was reported in a community in Arizona. Thus, the prevalence of this bacteria varies according to ethnicity, race, age, geographic region, and socioeconomic status [1]. This was confirmed by Mapel et al., who found that the pathogen is highly predominant in Hispanics [3].

There are numerous diagnostic methods for *H. pylori* infection testing, which can be invasive or non-invasive. The gold standard for diagnosis is the gastric biopsy, which is considered an invasive method. Therefore, the exploration of non-invasive tests has been implemented in the medical field. Non-invasive methods include the C13 and C14 urea breath tests, serology antigen tests, PCR, or antigen tests in stool samples.

Background

Results regarding the diagnostic accuracy of the antigen test in stool samples for *H. pylori* infection vary. In a study conducted by Gómez et al. in an Ecuadorian population of 86 patients with dyspepsia, the presence of *H. pylori* was studied using antigen tests in stool samples, serology, and histology; they determined sensitivity and specificity of 69.2% and 42.9% for antigen tests in stool samples, 64.2% and 47.7% for serology, and 42.5% and 69.2% for histology. It concluded that the sensitivity and specificity of antigen tests in stool samples and serology were low in the Ecuadorian population, where the prevalence of *H. pylori* is 89.53% [4]. However, in a study by Kinoshita da Silva et al., the antigen test in stool samples for *H. pylori* was validated. The study included 98 patients, and the test detected *H. pylori* antigen in 44 out of 50 positive patients, and they found a sensitivity of 88% [5].

Similarly, in another study conducted in 2015 by Calik et al., the accuracy of the antigen test in stool samples was compared with gastric biopsy. Out of the 122 patients in the study, 98 tested positive with the antigen test in stool samples, leading to the conclusion that this test has a sensitivity, specificity, and positive predictive value (PPV) of 92.45%, 81.25%, and 97.02%, respectively, demonstrating that the test is a good alternative that is rapid, cost-effective, and non-invasive [6].

Although there is evidence supporting the use of non-invasive tests, such as the antigen test in stool samples, as an alternative to invasive tests in other countries, the Dominican Republic lacks such information [1]. Therefore, it is necessary to determine the validity of this test to efficiently and reliably identify cases without increasing costs for patients in this specific population. The present study aims to define the diagnostic accuracy of the stool antigen test for *H. pylori* infection in the Dominican Republic.

## Materials and methods

This is an observational, retrospective, and cross-sectional study.

Population and sample

We reviewed the medical records of patients from a local gastroenterology clinic who underwent *H. pylori* stool antigen testing before undergoing an endoscopic biopsy in 2021.

Inclusion and exclusion criteria

Patients over 18 years old with an indication for endoscopy due to suspected *H. pylori* infection, who visited the gastroenterology clinic in 2021, were included. All those who underwent an antigen test in stool before endoscopy were included.

Patients with a history of treatment for *H. pylori* gastropathy, biliary pathologies, antibiotic use, anti-secretory medications, or bismuth within 30 days before diagnosis were not included. Pregnant women, those with gastric carcinoma, chronic kidney disease (CKD), and those with chronic liver disease were also excluded.

Data collection and data management

Information from patients who underwent endoscopy in 2021 was collected from the pathology laboratory and gastroenterology electronic medical records. Patients were identified with codes to preserve their identity. Microsoft Excel (Microsoft Corporation, Redmond, USA) was used to organize and store the data.

Variables

Sociodemographic and clinical variables were obtained from patients' medical records. The variables include age, gender, medical history, toxic habits, biopsy results, and *H. pylori *stool antigen test results.

Statistical analysis

Categorical variables are presented as absolute or relative frequencies. The normality of the variables was evaluated using the Shapiro-Wilk test. The STATA program (StataCorp LLC, College Station, Texas, USA) was used for data analysis. The chi-square test or Fisher's exact test was used for binary variables. Spearman's correlation was used for continuous variables. The t-test was applied for binary and continuous variables.

To determine the diagnostic values of the *H. pylori* antigen test in stool, false positives, false negatives, true positives, and true negatives will be determined. The corresponding calculations were performed to determine sensitivity, specificity, PPV, and negative predictive value (NPV) from the construction of a 2×2 table.

Ethical considerations

The ethical aspects of the research proposal were evaluated by an ethics committee: the National Council of Health Bioethics (CONABIOS).

## Results

Gastric biopsies were performed on 593 patients, and only 135 patients met the established criteria to be part of the study. The mean age was 45.28±17 years, and 54.81% were women (Table 1). The majority of patients had a positive result for *H. pylori* at the time of biopsy, accounting for 57.78% of the sample. However, only 38.46% had a positive result for the stool antigen test for *H. pylori *infection. Among the patients who showed no presence of *H. pylori* during the biopsy, 40.35% had a positive result for the stool antigen test.

**Table 1:** Sociodemographic factors and testing results for stool antigen and gastric biopsy

		Participants, n=135		Positive *H. pylori* on biopsy=78	Negative *H. pylori *on biopsy, n=57			p-value
Gender								
Feminine		74 (54.81%)		42 (56.76%)	32 (43.24%)			0.791
Masculine		61 (45.19%)		36 (59.02%)	25 (40.98%)			
Age		45.28±17		44±17	46±17			
*H. pylori *stool antigen test results								
Positive	71 (52.59%)		48 (61.54%)			23 (40.35%)	0.015	
Negative	64 (47.41%)		30 (38.46%)			34 (59.65%)		

The results showed that neither age nor gender influenced the results obtained in the biopsy and the stool antigen test for *H. pylori* infection (Table 1). However, there was a significant association between the biopsy results and the antigen test results. Based on the obtained results, it was observed that the stool antigen test for *H. pylori* infection has a sensitivity of 61.54% and a specificity of 59.65% (Table 2).

**Table 2:** Sensitivity and specificity of stool antigen test for H. pylori infection PPV, positive predictive value; NPV, negative predictive value

	* *Stool antigen test for *H. pylori* infection
Sensitivity	61.54%
Specificity	59.65%
PPV	67.60%
NPV	53.13%

## Discussion

The diagnosis of *H. pylori* infection through non-invasive tests has been the focus of the search for cost-effective detection methods. In these cases, the diagnostic values of these tests should be evaluated in each population. Within the studied population, 57.78% showed a positive result in the biopsy. From these data, the sensitivity, specificity, PPV, and NPV of the stool test for* H. pylori* infection were determined, by taking the gastric biopsy, which is the gold standard, as the reference value. It is worth noting that there was a statistically significant relationship between the stool antigen test and the gastric biopsy.

The study showed a sensitivity of 61.54%, similar to the results obtained by Bosch et al. who identified a sensitivity of 61% for the stool test for *H. pylori* infection in Florida [7]. It was also similar to the sensitivity identified by Sharbatdaran et al. in Iran (66%) [8]. These relatively low values can be explained by the characteristics of these populations, with Iran being a low-resource country and Florida being a state in the United States with a high rate of Latin American immigrants in which the prevalence may be higher. However, another study conducted in Iran by Tameshkel et al. showed a sensitivity of 80.4% [9]. Higher sensitivity values have been established in other regions, as described by Silva et al. and Calik et al. in Brazil, with values of 88% and 92.45%, respectively [5,6].

A meta-analysis conducted by Best et al. gathered information from 99 studies and estimated the sensitivity of the stool antigen test at a fixed specificity of 0.90 [10]. The result was 0.83 (95% CI 0.73 to 0.90), significantly higher than the one we detected (61.54%). This value was very similar to the one detected by Safarnezhad Tameshkel et al., who detected a sensitivity of 80.4%. A lower sensitivity, similar to our study, was determined by Sharbatdaran et al., who detected a value of 66% [11].

The specificity defined by the study was approximately 60%, contrary to the research published in different regions. However, it is consistent with the findings of Bosch et al., who described a specificity of 61% [7]. Other research found specificities of 81.25% by Calik et al., 85.71% by Tameshkel et al., 87.5% by Silva et al., and 91% by Sharbatdaran et al. [5,6,8,9]. The results in these studies justify the routine use of the stool antigen test for *H. pylori* infection as a post-treatment assessment. The determined specificity in the study could demonstrate a barrier to determining the efficacy of antibiotic therapy for pathogen eradication. Furthermore, there is high antibiotic resistance in the Latin American region regarding the use of *H. pylori* infection treatments, as described in a meta-analysis by Camargo et al.; treatment failure can be attributed to the deliberate use of antibiotic drugs [12].

The PPV was lower than that described in the literature, resulting in 67.60%. The authors recorded a PPV of 97% by Calik et al., 88% by Silva et al., 93% by Sharbatdaran et al., and 85.42% by Tameshkel et al. [5,6,8,9]. On the other hand, the NPV of the present study was 53.13%. Calik et al. and Sharbatdaran et al. also observed a relatively low NPV, obtaining 61.9% and 62%, respectively [6,8].

Limitations of the study include that the population analyzed was selected from a single health center, which reduces the external validity of the study by limiting the extrapolation of the information found in our patients to a larger population. However, the study provides an approach to the test's behavior in the evaluated population and contributes to future research with participants from different regions. As an observational study, it is only possible to establish associated factors and not a causal relationship. In addition, this research is retrospective and therefore has a greater risk of losing information about the study participants.

## Conclusions

The stool antigen test is an accessible, rapid, and minimally troublesome test for patients. This study analyzed and estimated the diagnostic efficacy in the Dominican population to determine its utility as a screening test. These tests are more reliable as their sensitivity increases. Conversely, they can be used to determine post-treatment eradication of the bacteria based on their specificity. This study determined low values for both sensitivity and specificity, making it pertinent to study alternative non-invasive methods, such as the breath test and serology antigen testing. Therefore, larger studies should be conducted, involving more non-invasive methods to better assess this matter in each population or region.
